# Supplementary material for: Gait and Balance Assessments with Augmented Reality Glasses in People with Parkinson’s Disease: Concurrent Validity and Test–Retest Reliability
Source: Sensors (Basel). 2024 Aug 24;24(17):5485. doi: 10.3390/s24175485 (PMC11398006; doi:10.3390/s24175485)
Supplement: Supplementary file 1 [file sensors-24-05485-s001.zip › Table S1.pdf]

Table S1. Concurrent validity statistics for FTSTS (sub-)durations (in s): between-systems absolute agreement statistics for (sub-)durations derived from AR and Kinect sternum and Spine-base data.

	**Mean ± SD**	**Mean ± SD**	**Bias (95% Limits of Agreement)**	***t*-statistics**	**ICC$_{(A,1)}$**	**Mean ± SD**	**Bias (95% Limits of Agreement)**	***t*-statistics**	**ICC$_{(A,1)}$**
	**AR**	**Kinect Sternum**				**Kinect Spine-base**			
**Completion duration**	11.95 ± 3.91	11.94 ± 3.75	-0.01 (-0.61 0.60)	$t(17) = 0.13$ $p = 0.900$	0.997	12.14 ± 3.71	0.19 (-0.35 0.73)	$t(17) = -2.86$ **p = 0.011**	0.996
**Sitting sub-duration***	0.69 ± 0.49	0.58 ± 0.45	-0.11 (-0.42 0.20)	$t(16) = 2.79$ **p = 0.013**	0.923	0.56 ± 0.40	-0.13 (-0.47 0.20)	$t(16) = 3.19$ **p = 0.006**	0.893
**Sit-to-stand sub-duration***	0.58 ± 0.19	0.66 ± 0.16	0.08 (-0.13 0.28)	$t(16) = -2.93$ **p = 0.010**	0.766	0.67 ± 0.16	0.08 (0.15 0.32)	$t(16) = -2.94$ **p = 0.010**	0.710
**Standing sub-duration***	0.34 ± 0.15	0.34 ± 0.17	-0.00 (-0.09 0.08)	$t(16) = 0.36$ $p = 0.725$	0.962	0.38 ± 0.23	-0.04 (-0.18 0.26)	$t(16) = -1.59$ $p = 0.131$	0.819
**Stand-to-sit duration***	0.62 ± 0.16	0.67 ± 0.18	0.06 (-0.08 0.20)	$t(16) = -3.34$ **p = 0.004**	0.858	0.65 ± 0.17	0.04 (-0.16 0.24)	$t(16) = -1.68$ $p = 0.113$	0.791

Significant biases are presented **in bold**. *One outlier participant (p4 in the supplementary data files) was excluded from the sub-duration results due to insufficient peaks in the Kinect time series only, preventing our peak-detection algorithm to accurately identify the frame numbers start and end indices.
