# Supplementary material for: Gait and Balance Assessments with Augmented Reality Glasses in People with Parkinson’s Disease: Concurrent Validity and Test–Retest Reliability
Source: Sensors (Basel). 2024 Aug 24;24(17):5485. doi: 10.3390/s24175485 (PMC11398006; doi:10.3390/s24175485)
Supplement: Supplementary file 1 [file sensors-24-05485-s001.zip › Table S2.pdf]

Table S2. Test-retest reliability: absolute agreement statistics for (sub-)durations (in s) derived from reference-systems data.

		Mean ± SD	Mean ± SD	Bias (95% Limits of Agreement)	*t*-Statistics	$ICC_{(A,1)}$
		**Stopwatch Trial 1**	**Stopwatch Trial 2**			
**FTSTS**	Completion duration	12.6 ± 4.2	12.0 ± 3.7	-0.57 (-3.67 2.54)	$t(19) = 1.60$, $p = 0.126$	0.915
**TUG**	Completion duration	10.4 ± 2.6	9.9 ± 3.2	-0.57 (-3.79 2.65)	$t(21) = 1.63$, $p = 0.119$	0.830
		**Kinect Trial 1**	**Kinect Trial 2**			
**FTSTS**	Completion duration	12.68 ± 4.19	12.29 ± 4.46	-0.39 (-3.18 2.40)	$t(11) = 0.95$, $p = 0.362$	0.946
	Sitting sub-duration	0.71 ± 0.45	0.68 ± 0.50	-0.03 (-0.32 0.26)	$t(11) = 0.78$, $p = 0.452$	0.953
	Sit-to-stand sub-duration	0.65 ± 0.19	0.65 ± 0.16	0.00 (-0.14 0.14)	$t(11) = -0.06$, $p = 0.848$	0.921
	Standing sub-duration	0.36 ± 0.13	0.35 ± 0.18	-0.01 (-0.26 0.23)	$t(11) = 0.35$, $p = 0.736$	0.713
	Stand-to-sit sub-duration	0.68 ± 0.17	0.69 ± 0.24	0.01 (-0.24 0.26)	$t(11) = -0.30$, $p = 0.768$	0.821
		**IMU Trial 1**	**IMU Trial 2**			
**TUG**	Turn 1 sub-duration	1.89 ± 0.81	1.86 ± 0.71	-0.03 (-1.00 0.95)	$t(19) = 0.25$, $p = 0.807$	0.793
	Turn 2 sub-duration	1.74 ± 0.60	1.61 ± 0.63	-0.13 (-0.65 0.38)	$t(19) = 2.28$, $\boldsymbol{p = 0.034}$	0.894

Significant biases are presented **in bold**.
